# Cardiovascular magnetic resonance of myocardial infarction after blunt chest trauma: a heartbreaking soccer-shot

**DOI:** 10.1186/1532-429X-11-39

**Published:** 2009-10-11

**Authors:** Hannibal Baccouche, Torsten Beck, Martin Maunz, Peter Fogarassy, Martin Beyer

**Affiliations:** 1 Medizinische Klinik II, Klinikum Kirchheim unter Teck, Kreiskliniken Esslingen, Germany

## Abstract

Cardiac injury occasionally occurs as a result of blunt chest trauma. Most cardiac complications in chest trauma are due to myocardial contusion rather than direct damage to the coronary arteries. However, traumatic coronary injury has been reported, and a variety of underlying pathophysiological mechanisms have been proposed. We present a 26 year old patient presenting with an acute coronary syndrome as a consequence of a soccer-shot impact to the chest. CMR showed apical inferior infarction, as well as multiple small septal lesions which were presumed to have resulted from embolization. The culprit lesion was a proximal 75% LAD stenosis with a prominent plaque-rupture and thrombus-formation, and the distal LAD was occluded by thromboembolic material.

## Background

In blunt chest trauma patients, it is important to consider myocardial injury, which is mostly the result of myocardial contusion. The consequences of contusion include ECG-changes, arrhythmia and necrotic damage of the heart muscle. Direct damage to the coronary arteries however is a rare finding. Data published on the prevalence of cardiac affection in chest trauma and its further sub-categorization is sparse. Maenza and colleagues performed a meta-analysis on cardiac complications in blunt cardiac trauma including more than 4600 patients. The prevalence of cardiac complications varied between 2.6% and 4.5%. Myocardial infarction or complications assuming infarction occurred only in a minority of the patients (between 5 and 7%) [1]. Christensen et al. identified 77 published cases of acute myocardial infarction in blunt chest trauma. The LAD was the most commonly affected vessel, followed by the RCA and RCX. Mechanisms of coronary-damage included dissection, plaque-rupture with thrombosis and/or thromboembolism, spasm, vessel rupture and epicardial hematoma with external compression. Patients suffering from a traumatic injury of a coronary artery were considerably younger than the normal age of presentation of coronary disease (> 80% were less than 45 years old). The most common trauma causing myocardial infarction came from road traffic accidents, followed by sporting accidents including soccer [2].

The time interval from injury to coronary vessel occlusion showed a highly variable course reaching from immediate onset to a delay of several weeks [3]. Diagnostic approaches for cardiac involvement in chest trauma patients comprise cardiac biomarkers (creatine kinase and troponin), ECG-testing and echocardiography. Computed tomography has been used both for visualizing a damaged coronary vessel and detecting regional myocardial perfusion defects [4]. CMR has been successfully used in chest trauma [5,6], although its exact role in assessing cardiac involvement has not yet been clearly defined.

## Case presentation

**Figure 1:**
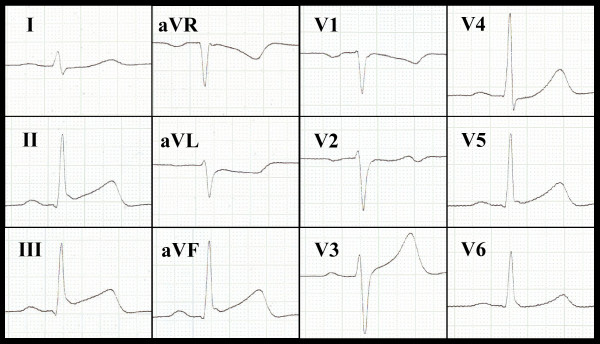
**Twelve lead ECG demonstrating a classical picture of inferiorly located STEMI with ST-segment elevation in the inferior leads (II, III, aVF) and ST-segment depression in lead I and aVL**.

A 26 year old male patient was referred to our hospital with an acute coronary syndrome. He had experienced blunt chest trauma while playing soccer, taking a high velocity shot on the chest. The trauma raised an intense episode of retrosternal chest pain. After a short pain-free interval more intense chest-pain recurred leading to emergency hospital admission, after which acute inferior ST-segment-elevation myocardial infarction was diagnosed. (Figure 1, full motion images can be viewed at ).

With the exception of a family history of myocardial infarction, no other cardiovascular risk factors were present. Furthermore, there was no evidence indicating that a viral prodrome suggestive of myocarditis was present.

**Figure 2:**
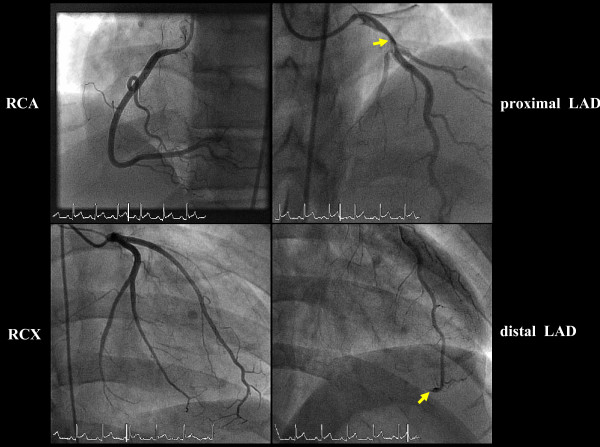
**Invasive coronary angiography demonstrates single vessel coronary artery disease in the LAD with a proximal 75% stenosis showing a prominent plaque-rupture with thrombus-formation and distal occlusion**. The LAD is wrapping around the LV apex. The other coronaries (selective intubation) are without significant stenosis.

Cardiac catheterization revealed single vessel-coronary-artery-disease with a proximal 75% left anterior descending artery (LAD) stenosis showing prominent plaque rupture with thrombus formation. There was thromboembolic distal LAD occlusion of the prominent LAD vessel, which was also supplying the apical inferior portion of the heart by wrapping around the LV apex, therefore leading to inferior infarction. (Figure 2, full motion images can be viewed at ).

Bare metal stenting of the proximal lesion was successfully performed (no remaining stenosis). Due to the localisation of distal thromboembolism, thrombus aspiration was no option. Medical therapy with glycoprotein IIb/IIIa-inhibitor, clopidogrel, aspirin and heparin was administered and distal flow was able to be restored. Serial serum creatinekinase measurements revealed a maximum CK-rise to 1950 U/l.

**Figure 3:**
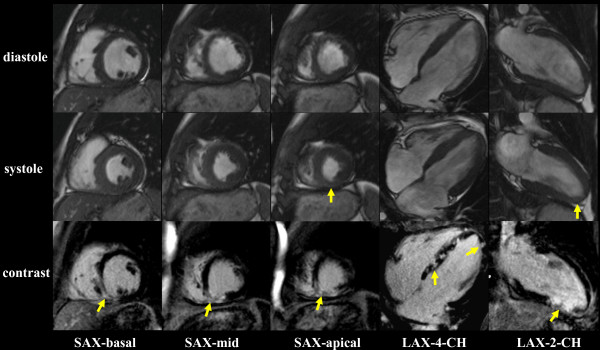
**Multiple CMR short and long axis views, demonstrating inferior apical akinesia in the SSFP cine sequences**. Contrast images show transmural LGE in the inferior apical region as a consequence of distal LAD occlusion and numerous focal patches of intramural LGE due to multiple embolic infarctions via the septal branches.

CMR was performed to assess myocardial viability (1.5 T Avanto, Siemens Medical Systems, Erlangen, Germany). Cine images were acquired using fast gradient echo steady-state free precession sequences (SSFP) demonstrating inferior apical akinesia (Figure 3). Systolic left ventricular function was mildly impaired (LVEDV 166 ml, LVEF 58%). Ten minutes after injection of 0.2 mmol/kg Gd-DTPA (Bayer Schering, Germany), CMR was performed using inversion-recovery-gradient-echo-technique, adjusting inversion time to null normal myocardium. Late gadolinium enhancement (LGE) was present in the inferior apical region of the heart with mainly transmural extent. Additional unexpected focal patches of intramurally distributed LGE were also seen in the interventricular septum, most likely correlating with multiple small embolic infarctions via the septal branches of the LAD. (Figure 3, full motion images can be viewed at ).

The further clinical course of the patient was uneventful. At 2 months of follow up, CMR showed normal global left ventricular function and the myocardial scarring had clearly shrunk. The patient was not suffering from any cardiac symptoms and was able to enjoy sports again without limitations.

## Conclusion

Acute myocardial infarction is a rare entity in chest trauma. It may result from contusion or damage to the coronary arteries. In the latter, plaque rupture, thrombus formation, coronary artery dissection, focal spasm and other rare mechanisms have to be considered. As clinical management changes if coronary perfusion is impaired, it is crucial to identify those patients in need for invasive workup including cardiac catheterization und angioplasty. Diagnostic standard approaches to cardiac injury in chest trauma patients by ECG, lab-testing and echocardiography can be supplemented by tomographic imaging as demonstrated by CMR.

## Consent

Written informed consent was obtained from the patient for publication of this case report and any accompanying images. A copy of the written consent is available for review by the Editor-in-Chief of this journal.

## Competing interests

The authors declare that they have no competing interests.

## Authors' contributions

HB conceived and designed this case report, was involved in data acquisition (performed the CMR scan) and interpretation and drafted both the text and figure file. TB designed this case report, was involved in data acquisition (performed cardiac-catheterization and PCI on the patient), carried out literature review and drafted the image file. MM was involved in data acquisition (cardiac catheterization, literature) and clinical management of the patient, made contributions to drafting both text and image files and the webpage and critically reviewed the documents. PF was involved in data acquisition, interpretation and patient management, contributed to drafting of text and images, designed the webpage and critically reviewed the documents. MB was involved in data acquisition and interpretation and contributed to drafting of text, images and webpage and critically reviewed all documents. MB supervised the clinical management of the patient and finally this case report. All authors take responsibility for the entire content of this report and have read and approved the submission of this manuscript.
